# Editorial: Neuromodulation and neural technologies for sight restoration

**DOI:** 10.3389/fncel.2023.1304872

**Published:** 2023-10-17

**Authors:** Maesoon Im, Günther M. Zeck, Leanne Lai Hang Chan, Diego Ghezzi, Shelley I. Fried

**Affiliations:** ^1^Brain Science Institute, Korea Institute of Science and Technology (KIST), Seoul, Republic of Korea; ^2^Division of Bio-Medical Science & Technology, KIST School, University of Science and Technology (UST), Seoul, Republic of Korea; ^3^KHU-KIST Department of Converging Science and Technology, Kyung Hee University, Seoul, Republic of Korea; ^4^Institute of Biomedical Electronics, TU Wien, Vienna, Austria; ^5^Department of Electrical Engineering, City University of Hong Kong, Kowloon, Hong Kong SAR, China; ^6^Ophthalmic and Neural Technologies Laboratory, Department of Ophthalmology, Hôpital Ophtalmique Jules-Gonin, Fondation Asile des Aveugles, University of Lausanne, Lausanne, Switzerland; ^7^Boston VA Medical Center, Boston, MA, United States; ^8^Department of Neurosurgery, Massachusetts General Hospital, Harvard Medical School, Boston, MA, United States

**Keywords:** artificial vision, sight restoration, neuromodulation, neural technologies, visual prosthesis, retinal prosthesis

The ability to restore vision to the blind with artificial stimulation remains one of the tantalizing goals of neural engineering and many new technological developments offer ongoing advances in support of this effort. Nevertheless, clinical test results remain sub-optimal, i.e., most approaches continue to elicit light percepts (phosphenes) but the reliable creation of more complex visual patterns remains elusive. In this Frontiers Research Topic entitled “*Neuromodulation and neural technologies for sight restoration*,” 11 Original Research articles, 3 Mini Review and one Perspective describe efforts ranging from an in-depth analysis of clinical test results focused on better understanding existing limitations to development of novel strategies for driving the retina to fundamental modeling and physiological studies focused on the development of more effective stimulation strategies.

In the first study, Yücel et al. examined the factors underlying high thresholds and poor spatial resolution in users of the Argus II epiretinal implant and found that inadvertent activation of passing axons, lift of the device off the retinal surface, and retinal damage all played a role. Although the Argus II is no longer commercially available, the analysis and discussion of the 3 individual components provide insights that will be relevant to many other devices.

Current research focuses on therapeutic strategies to restore vision in retinal degeneration (RD) patients. However, there is limited investigation into neural activities in the brain following retinal electrical stimulation. Additionally, the neuromodulatory changes resulting from prolonged electrical stimulation in the brains of retinitis pigmentosa (RP) patients have not been studied. Agadagba et al. studied the effects of transcorneal retinal electrical stimulation on functional and directional connectivity in visual and non-visual brain cortices of RD mice. The results showed that transient stimulation did not significantly alter brain connectivity, but 1-week post-stimulation revealed increased theta-gamma coupling and enhanced coherence and connectivity in theta, alpha, and beta oscillations. Notably, 2-weeks post-stimulation demonstrated long-lasting enhancements in network coherence and connectivity between visual and non-visual cortices. These findings suggest that transcorneal retinal electrical stimulation can modulate brain connectivity and may have implications for higher cortical functions.

Rapid fading of artificial visual percepts has been reported in clinical studies. To explore this, Li et al. investigated how desensitization of mouse retinal ganglion cells (RGCs) can be mitigated by changing stimulation parameters. They reported the RGC desensitization is not related to stimulus amplitude but stimulation frequency, suggesting the fading may be partially addressed by optimizing the stimulation conditions. In past retinal prosthetic studies, the various genotypes of retinal degenerative diseases have not been well considered. For example, more than 80 different genes have been identified to cause RP. Roh et al. compared phenotypes of the retinas of *rd8* and *rd10* mice which have *Crb1* and *Pde6b* mutations, respectively. They also systematically examined electrically-evoked network-mediated spiking responses of RGCs (Im and Fried, 2015; Roh et al., 2022) of the *rd8* retinas by first classifying the types of RGCs into non-direction selective ON, non-direction selective OFF, or direction selective ON-OFF (Im and Fried, 2016; Otgondemberel et al., 2021). Then, they compared the electric responses of those cells with their own visually-evoked responses and found the correlation levels of those two responses (i.e., electric and visual responses) are differentially altered even at the early stage of degeneration caused by the *Crb1* gene. Spiking patterns of the electrically-evoked responses of *rd8* retinas seem to be similar to those of early-stage *rd10* retinas (Yoon et al., 2020).

Ahn et al. study retinal ganglion cell activity in photoreceptor-degenerated *ex vivo* mice and macaque *ex vivo* retinae upon electrical stimulation. They identified a spatially extended activation (>800 μm) to short and strong electrical stimulation of the RD retina. This activity was mediated by stimulation of the electrically coupled bipolar-amacrine network. The authors suggest that such wide-spread activation may have limited the outcome of retinal prosthetics. Indeed, the use of brief, strong stimulus currents may represent a challenge for photoreceptor-degenerated retinae, where a more localized activation has been reported for lower current amplitudes in previous studies (Ryu et al., 2017; Corna et al., 2021). Future work may identify if the aberrant activity presynaptic to the RGCs across large retinal portions persist if time-varying, non-stationary stimuli with variable amplitude (Chenais et al., 2021) or waveforms (Höfling et al., 2020) are applied.

The degree of degeneration and preserved responsiveness is of importance in restoration of artificial vision. Cha et al. investigated stage-dependent changes of visual function and electrical responsiveness in *rd10* mice. The authors report light-induced activity in RGCs in the *ex vivo* retina up to postnatal day 70, and interestingly, a longer preservation of the ON response. Previously, it was thought that OFF-type RGCs would survive longer (Stasheff, 2008) in the rod-degenerated mouse retina. Future work may clarify if preservation of RGC classes is specific to certain strains. The study by Cha et al. also shows that for certain pulsatile stimuli presented by one electrode in epiretinal configuration activates only a small portion of RGCs. The spatial confinement, however, remained unclear.

High resolution discrimination of grating-like electrodes has been reported by Cojocaru et al.. They applied electrical stimulation in rod-photoreceptor degenerated retinae in epiretinal configuration using high-density electrode arrays. Here, stimulation was performed using sinusoidal waveforms presented continuously at 40 Hz, i.e., a stimulus duration of 25 ms. The authors demonstrated localized activation of RGCs with fine electrode gratings, down to 32 μm. They demonstrated avoidance of axonal stimulation using these waveforms—a long lasting, open question in retinal prosthetics. In agreement with previous work using point-like electrodes it appears that sinusoidal waveforms locally stimulate ganglion cells. Cojocaru et al. furthermore compared their results in the same retina with optogenetic stimulation of either bipolar cells transduced with ChR2 or with optogenetic stimulation of RGCs transduced with ChR2. Interestingly, they could not identify any significant differences regarding the spatial resolution, which was even higher than with electrical stimulation. Based on support vector machine classifier Cojocaru et al. estimated an optimal resolution with optogenetic stimulation of ~3 cycles/degree, corresponding to 20/200, in line with previous *ex vivo* work (McGregor et al., 2020; Gauvain et al., 2021; Reh et al., 2022).

A major concern using optogenetics for vision restoration in the retina had been the high light intensity. and efficacy of coupling. The concern with high light intensities has been solved by using optogenetic transducers coupled to G-protein receptors (van Wyk et al., 2015), with mGluR6 expressed in the retina by ON bipolar cells. Specific targeting of chimeric transducers, i.e., OptoGPCR chimeras between light gated G-protein coupled receptors (opsins) and ligand-gated G-protein coupled receptors. The study by Leeman and Kleinlogel sheds light on the expression efficacy of these chimeras.

Advancement in interfacing electronics would be essential for the enhanced performance of prosthetic systems. In this Research Topic, Hayashida et al. presented an advanced multichannel stimulation module for intracortical micro-stimulation in preclinical animal studies. They introduced a scalable 64-channel stimulation module, allowing for a deeper understanding of the relationship between the spatiotemporal patterns of stimuli and the resulting neural responses through pre-clinical animal studies. An application-specific integrated circuit (ASIC) chip was designed and implemented as the primary current output device, while a field-programmable gate array (FPGA) served as the controller. *In-vivo* experiments demonstrated the stimulation effect with a temporal resolution in the millisecond range. The multichannel stimulation module enabled control over not only the shape of each biphasic current pulse but also the degree of charge imbalance and the temporal order of pulse among multiple channels. These features are considered valuable for intracortical micro-stimulation in preclinical animal studies.

Computational approaches also can deepen our understanding of neuromodulation effects as well as expedite the development processes. For example, Sajedi et al. used computer simulation to examine the blocking phenomenon which may be caused by electric stimulation above an upper threshold of nerve cells. They investigated the effect of overstimulation for layer 5 pyramidal cells and alpha RGCs and suggested the importance of an appropriate stimulation window for the reliable activation of target neurons for neural prosthetic applications. Also, Rahmani and Eom used computational analysis of an organic solar cell that has spherical gold nanoparticles (AuNPs) for retinal prosthetic applications. Their analysis indicated the incorporation of AuNPs can enhance the photoconversion efficiency, thereby lowering the required light intensity for photovoltaic activation of retinal neurons. These studies suggest the efficiency of the computational approaches which can be experimentally verified in the follow-up studies.

The 3 Mini Reviews recapitulate three different topics in the field of visual prosthetics. In the first one by Wu et al., implanted devices that use a chemical stimulus (instead of electrical or optical ones) to reactivate the retina are discussed including the components of a chemical retinal prosthesis, the potential spatial and temporal resolution, and the possibility of achieving naturalistic stimulation. The biocompatibility and excitotoxicity of these kinds of implants are reviewed as well. The next logical step for chemical retinal prostheses is validation in *in-vivo* animals. In the second review article of Badadhe et al., the authors summarized the current status of ultrasound stimulation, a modality for sight restoration that is much less invasive than most existing approaches. Although several studies have demonstrated somewhat promising results of the acoustic prosthesis, Badadhe et al. pointed out that the working principle behind this modality is yet to be completely understood for more accurate neuromodulation.

The third review offered a new aspect of artificially-created neural activity for vision restoration by recapitulating computational neuroscience approaches to quantify neural information. In the work of Kim et al., the authors claim that there have been very few efforts such as the study of Kang et al. (2021) to understand the quality of neural spiking activities in the field of visual prosthetics, particularly in terms of the amount of neural information generated by any form of artificial stimulation. They briefly introduced the two different methods (i.e., direct and reconstruction methods) that can calculate information rates from the recorded spike trains. Lastly, in a Perspective article, Grani et al. argued that closed-loop stimulation strategies can substantially improve the effectiveness of cortical visual prostheses. They have succinctly summarized previous examples of closed-loop strategies applied in various neural prostheses and several challenges to be overcome in future research.

Taken together, the 15 articles in the present Research Topic describe many of the aspects that need to be considered as new and ongoing efforts strive to improve performance of retinal and other visual prostheses. While progress toward the goal of near-normal vision with an implant remains limited, such efforts continue to offer hope to the millions of people who are blind.

## Author contributions

MI: Writing—original draft, Writing—review and editing. GZ: Writing—original draft, Writing—review and editing. LC: Writing—original draft, Writing—review and editing. DG: Writing—original draft, Writing—review and editing. SF: Writing—original draft, Writing—review and editing.
